# Efficacy of the herbal medicine Chotosan following treatment with Western medications for migraine accompanied by tension-type headache

**DOI:** 10.3389/fneur.2025.1697333

**Published:** 2025-12-09

**Authors:** Takafumi Tanei, Satoshi Yamashita, Satoshi Maesawa, Yusuke Nishimura, Tomotaka Ishizaki, Yoshitaka Nagashima, Yoshiki Ito, Miki Hashida, Takahiro Suzuki, Shun Yamamoto, Toshihiko Wakabayashi, Ryuta Saito

**Affiliations:** 1 Department of Neurosurgery, Nagoya University Graduate School of Medicine, Nagoya, Aichi, Japan; 2 Department of Specialized Headache Outpatient, Nagoya Garden Clinic, Nagoya, Aichi, Japan; 3 Department of Neurosurgery, National Hospital Organization, Nagoya Medical Center, Nagoya, Aichi, Japan

**Keywords:** migraine, tension-type headache, Chotosan, herbal medicine, Kampo

## Abstract

**Background:**

Chotosan is herbal medicine that is effective for tension-type headache (TTH). This study aimed to evaluate the efficacy of Chotosan following treatment with Western medications for migraine accompanied by TTH.

**Methods:**

This was a single-center, single-arm, and retrospective observational study. Chotosan was prescribed in the following situations: responded to Western medications but effects weakened; could not continue taking Western medications due to side effects; responded to Western medications but still had frequent headaches; and refused to take antidepressants. The primary endpoint was to determine whether monthly headache days (MHDs) were decreased 1 month after starting Chotosan treatment. The efficacy of Chotosan was defined as meeting two criteria: MHDs decreased by 33% or more, and the patient was willing to take and actually continued taking the medication. The efficacy rate of Chotosan, changes in monthly migraine days, changes in monthly triptan and nontriptan tablet usage, and incidence of side effects were also evaluated.

**Results:**

Of 1,030 migraine patients, 51 patients were included in the analysis. Chotosan significantly reduced MHDs from a median of 19.0 [14.8–25.8] days to 14.0 [7.8–23.8] days, with an efficacy rate of 52.9%. Median monthly migraine days also decreased significantly from 3.0 [0.0–6.0] days to 1.0 [0.0–3.8] days. The median amounts of triptan and non-triptan tables decreased significantly from 6.0 [2.0–9.8] tablets to 4.0 [1.0–7.0] tablets, and median 10.0 [0.8–17.3] tablets to 5.0 [0.8–18.5] tablets, respectively. The incidence of side effects was 3.9%.

**Conclusion:**

For patients with migraine accompanied by TTH who have not responded adequately to Western medications, Chotosan is a treatment option worth considering.

## 1 Introduction

The primary headache disorders are primarily migraine and tension-type headache (TTH), each of which now has up-to-date comprehensive reviews (1, 2). Migraine is a common disabling neurological disorder that is estimated to affect more than one billion people worldwide (3). Many migraine patients find relief from their headache symptoms through treatment with Western medications in line with the Clinical Practice Guideline for Headache (4). Western medications for migraine are divided into two categories: acute medications that suppress migraine attacks, and prophylactic medications that reduce the frequency and intensity of headaches. Conventional acute medications include triptans, acetaminophen, and nonsteroidal anti-inflammatory drugs (NSAIDs). Recently approved acute medications include lasmiditan, ubrogepant, and rimegepant, of which lasmiditan is available in Japan, and its efficacy has been reported (5). Oral prophylactic medications include anticonvulsants, antidepressants, calcium channel blockers, and beta-blockers. Furthermore, the recent development of anti-calcitonin gene-related peptide monoclonal antibodies (CGRP-mAbs) has led to dramatic improvement in preventive treatment (6–9). However, treatment with Western medications may not be effective, may become less effective over time, or may cause side effects that make it impossible to take the medications.

In today’s world, in which the use of digital devices has become indispensable in our work and daily lives, many people suffer from chronic neck and shoulder stiffness, which can lead to TTH. Significant correlations have been found between the time spent on computer or smartphone screens and increased frequency and severity of headaches (10–12). A recent study found that internet use was especially associated with TTH severity (13). In addition to using digital devices, a long time sitting in urban life and high work pressure can also cause TTH (14). Therefore, the number of patients with migraines accompanied by TTH that causes a persistent feeling of heaviness in the head is increasing. In patients with migraine accompanied by TTH, symptoms of TTH may persist despite treatment with CGRP-mAbs, leaving options limited to acetaminophen, NSAIDs, and antidepressants (15).

In Japan, the herbal medicines known as Kampo medicines are used to treat a variety of clinical conditions, including headaches, cold symptoms, stomach aches, and insomnia. Kampo medicines are made by combining various herbal medicines. There are over 100 types of Kampo medicines, and the most famous one is Kakkonto. The herbal medicines are mainly used in Asian countries such as Japan, Korea, and China. The Japanese Clinical Practice Guideline for Headache introduces Kampo medicines such as Goreisan, Goshuyuto, Kakkonto, Keishininjinto as treatments for migraine (4). Goreisan (16–18) and Goshuyuto (16, 17, 19) are herbal medicines that are effective for migraines. Chotosan is a herbal medicine that is effective for TTH, especially in middle-aged and elderly patients who have high blood pressure (16, 20). Chotosan is prepared using 10 herbs and gypsum. The herbal ingredients include dried *Uncaria* hook, dried *Ophiopogon* tuber, dried *Pinellia* tuber, dried citrus peel, dried *Panax ginseng*, dried *Poria cocos*, dried *Saposhnikovia divaricata*, dried chrysanthemum, and dried liquorice.

Although herbal medicines have been used empirically for migraine and TTH with good results in clinical practice, one of the major problems in the field of herbal medicines is that there is little evidence, and even if there is, much of the evidence has been published in languages other than English (16–20). Therefore, the aim of this study was to scientifically evaluate the efficacy of the herbal medicine Chotosan following treatment with Western medications for migraine accompanied by TTH. This is an open study without blind randomization.

## 2 Materials and methods

### 2.1 Study design

This was a single-center, retrospective, real-world study of patients with migraine accompanied by TTH prescribed the herbal medicine Chotosan. The patients were recruited from the specialized headache outpatient clinic at Nagoya Garden Clinic from May 2022 to March 2025. All patients had a diagnosis of migraine according to the International Classification of Headache Disorders 3 criteria (21). Patients first underwent magnetic resonance imaging to exclude intracranial diseases, and then medical treatment for migraine was started. Diagnosis and treatment were performed by a neurosurgeon specializing in the field of pain and headaches (T.T.). The treatment policy for migraine was as follows. Triptans were mainly used as acute medication. Lasmiditan or NSAIDs were used when triptan alone was not sufficiently effective or could not be used due to side effects (5). In cases with frequent headaches, oral prophylactic medications such as anticonvulsants, antidepressants, calcium channel blockers, and beta-blockers were prescribed based on the attending physician’s experience. If patients agreed to start CGRP-mAb treatment, one of three CGRP-mAb drugs was selected through patient-physician discussion in the clinical setting (9).

Chotosan was prescribed to patients with migraine accompanied by TTH in the following cases: those who responded to treatment with Western medications but the effects weakened; those who could not continue taking Western medications due to their side effects; those who responded to treatment with Western medications but still had frequent headaches; and those who refused to take antidepressants as oral prophylactic medications for TTH symptoms. The dosage of Chotosan was 7.5 g per day, divided into three doses before each meal. Because the herbal medicines are a bitter powder, if the patient did not wish to take it three times a day, the daily dose was 5.0 g in two divided doses.

This study was approved by the Ethics Review Committee of Nagoya University Graduate School of Medicine (approval number 2024-0251). Since this study was noninvasive, the Ethics Review Committee of Nagoya University Graduate School of Medicine waived the requirement for written, informed consent from patients, but the opt-out method was adopted in accordance with the Japanese ethics guidelines. This research was completed in accordance with the Declaration of Helsinki as revised in 2013.

### 2.2 Data collection

Demographic data (age, sex, onset years of migraine, family history of headache, history of psychiatric disorders, migraine with aura, classification of migraine, use of oral prophylactic medications and type, and use of CGRP-mAb injections) were collected retrospectively. Migraine was classified into four types: episodic migraine (EM), high-frequency episodic migraine (HFEM), chronic migraine (CM), and medication overuse headache (MOH). Monthly headache days (MHDs) of 0–7 days were defined as EM, and 8–14 days as HFEM. Most patients kept headache diaries to record the number of headaches, migraines, and acute medications usage. During the month before and after starting Chotosan, MHDs, monthly migraine days (MMDs), monthly amount of triptan and non-triptan tablet usage, and side effects were collected from their headache diaries or electronic medical records.

### 2.3 Assessments and statistical analysis

The primary endpoint of this study was the decrease in MHDs in the month after starting Chotosan treatment compared with the month before. In the present study, Chotosan was defined as effective if it met two criteria: MHDs were reduced by 33% or more, and the patient was willing to take and actually continued taking the medication. The following secondary endpoints were examined during the same evaluation period: the efficacy rate of Chotosan, MMD changes, changes in monthly amounts of triptan and nontriptan tablets, and the incidence of side effects. Based on the definition of efficacy of Chotosan, patients were divided into the effective group and the ineffective group, and differences in background factors between the two groups were examined. As an additional supplementary analysis, the efficacy rate of Chotosan was calculated, with effective defined as a 50% or greater reduction in MHDs. Furthermore, MHDs, MMDs, and monthly amounts of triptan and non-triptan tablets after 3 months of treatment were compared with those before treatment and after 1 month of treatment, respectively.

The Shapiro–Wilk test was performed on each variable to check whether each measure followed a normal distribution. Next, for categorical variables, the Wilcoxon signed-rank test was performed to confirm the effect of treatment. The absolute change values and 95% confidence intervals (CIs) for MHDs and MMDs were calculated. Mann–Whitney’s U test was used for comparisons between the effective and ineffective groups. Fisher’s exact test was used to evaluate categorical variables for items with a small sample size. The Bonferroni method was used to adjust for multiple comparisons. Significance was set at *p* < 0.05. All statistical analyses were performed using EZR (Saitama Medical Center, Jichi Medical University, Saitama, Japan), a graphical user interface for R (The R Foundation for Statistical Computing, Vienna, Austria), a modified version of R commander designed to add statistical functions frequently used in biostatistics (22).

## 3 Results

### 3.1 Participants’ demographic characteristics

**Figure 1:**
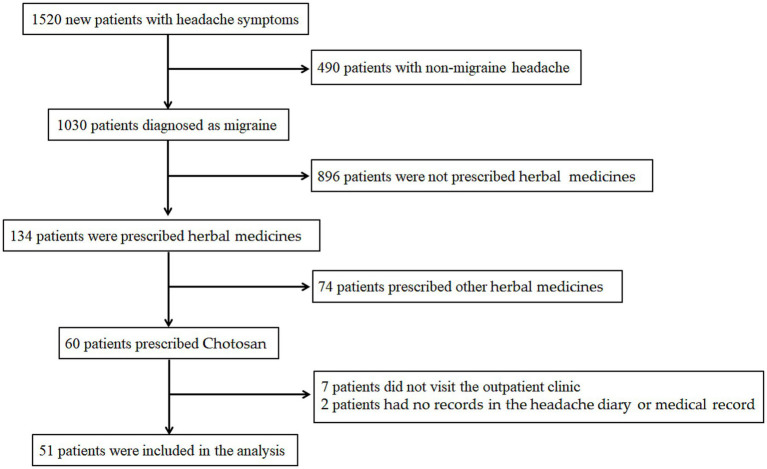
Flowchart showing patient selection.

From May 2022 to March 2025, 1,520 new patients with headache symptoms visited the specialized headache outpatient clinic, of whom 1,030 (67.8%) were diagnosed as having migraine. The migraine classification for all patients included EM (*n* = 613, 59.5%), HFEM (*n* = 196, 19.0%), CM (*n* = 116, 11.3%), and migraine with MOH (*n* = 105, 10.2%). Of all patients with migraine, 155 (15.0%) were receiving CGRP-mAb treatment. Of the 1,030 migraine patients, 134 (13.0%) were prescribed some kind of herbal medicines during the study period. Of the patients who were prescribed herbal medicines, 60 were prescribed Chotosan. Seven patients who did not visit the outpatient clinic after being prescribed Chotosan and two patients who did not have records in the headache diary or electronic medical record were excluded. Therefore, 51 patients were included in the analysis of this study (Figure 1). The clinical characteristics of the eligible patients are shown in Table 1. The mean age was 40.6 ± 10.8 years, with females accounting for 88.2% (45/51). The onset ages of migraine were teens and younger (*n* = 23, 45.1%), 20s (*n* = 18, 35.3%), 30s (*n* = 9, 17.6%), and 40s and older (*n* = 1, 2.0%). Thirty-two patients had a family history of headaches (62.7%), and 8 patients had a history of psychiatric disorders (15.7%). Migraine with aura was present in 19 patients (37.3%). The migraine classification of patients included in the analysis included EM (*n* = 13, 25.5%), HFEM (*n* = 16, 31.4%), CM (*n* = 6, 11.8%), and migraine with MOH (*n* = 16, 31.4%). At baseline before taking Chotosan, median MHDs and MMDs were 19.0 [interquartile range (IQR), 14.8–25.8] days and 3.0 [IQR, 0.0–6.0] days, respectively, and the median amounts of triptan and non-triptan tablets were 6.0 [IQR, 2.0–9.8] tablets and 10.0 [IQR, 0.8–17.3] tablets, respectively. Forty-three patients (84.3%) were taking oral prophylactic medications. The types of oral prophylactic medications (including overlaps) included anticonvulsants (38), antidepressants (30), calcium channel blockers (15), and beta-blockers (0). Nineteen patients were receiving CGRP-mAb treatment (37.3%).

**Table 1:** Demographic and clinical characteristics of the patients.

Characteristics	*n* = 51
Age (years), mean ± SD	40.6 ± 10.8
Sex, female; *n* (%)	45 (88.2%)
Onset years; *n* (%)	
Teens and younger	23 (45.1%)
20s	18 (35.3%)
30s	9 (17.6%)
40s and older	1 (2.0%)
Family history of headaches; *n* (%)	32 (62.7%)
History of psychiatric disorders; *n* (%)	8 (15.7%)
Migraine with aura; *n* (%)	19 (37.3%)
Classification of migraine	
EM	13 (25.5%)
HFEM	16 (31.4%)
CM	6 (11.8%)
Migraine with MOH	16 (31.4%)
Headache and acute medication	
MHD	19.0 [14.8–25.8]
MMD	3.0 [0.0–6.0]
Triptan (tablets/month)	6.0 [2.0–9.8]
Non-triptan (tablets/month)	10.0 [0.8–17.3]
Oral prophylactic available	43 (84.3%)
Types of prophylactic medications^*^	
Anticonvulsants	38 (74.5%)
Antidepressants	30 (58.8%)
Calcium channel blockers	15 (29.4%)
Beta blockers	0
CGRP-mAb treatment	19 (37.3%)

Data are expressed as median [interquartile ranges]; CGRP-mAb, anti-calcitonin gene-related peptide monoclonal antibody; CM, chronic migraine; EM, episodic migraine; HFEM, high frequency episodic migraine; MHD, monthly headache days; MMD, monthly migraine days; MOH, medication overuse headache; *n*, number; SD, standard deviation; *Including overlaps.

### 3.2 Efficacy and side effects of Chotosan

**Figure 2:**
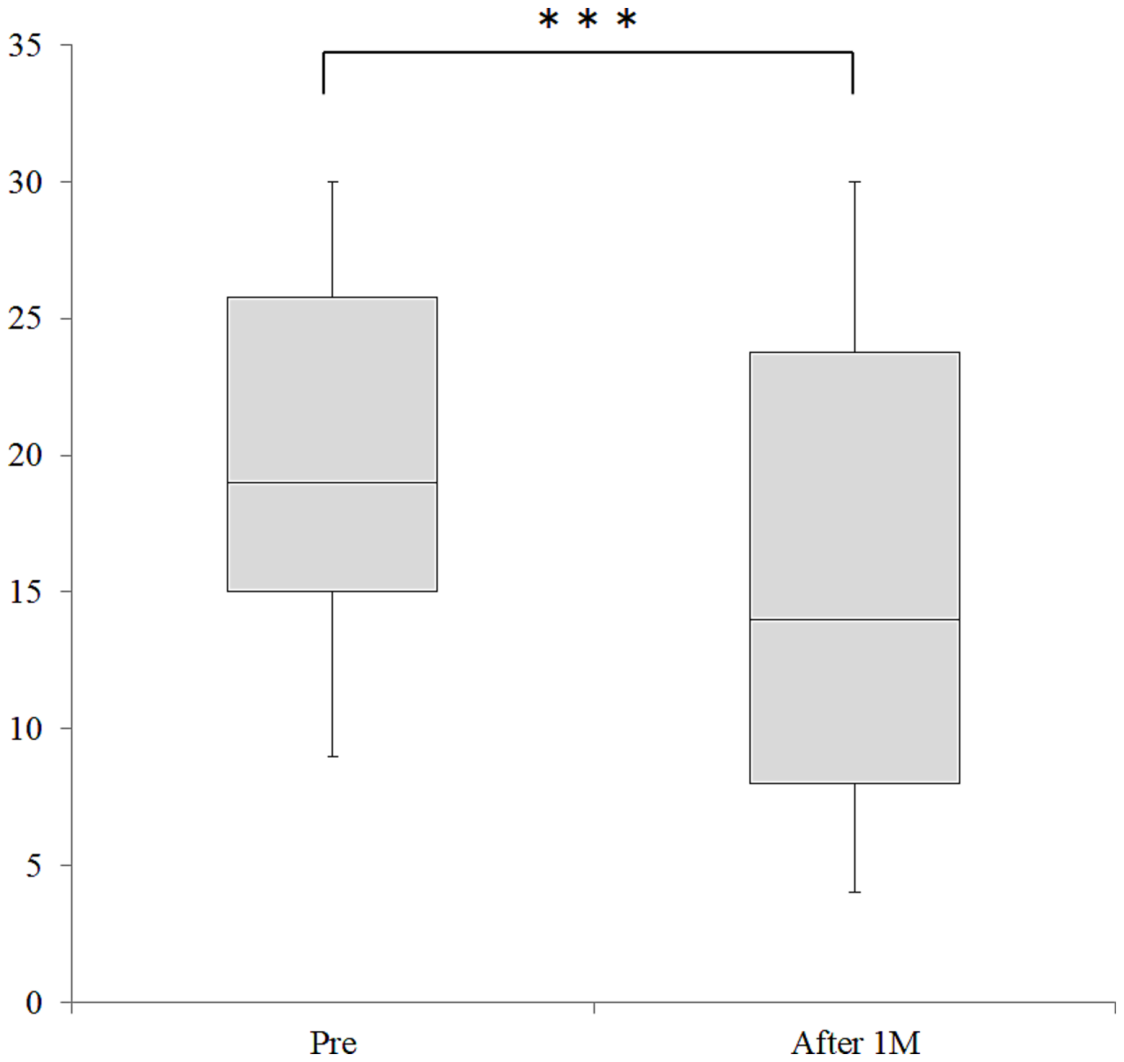
Changes in MHD before and after Chotosan treatment. The boxes represent the 25 and 75% interquartile ranges, and the line inside the box represents the median. ***Significant differences compared with baseline, *p* < 0.001; M, month.

**Figure 3:**
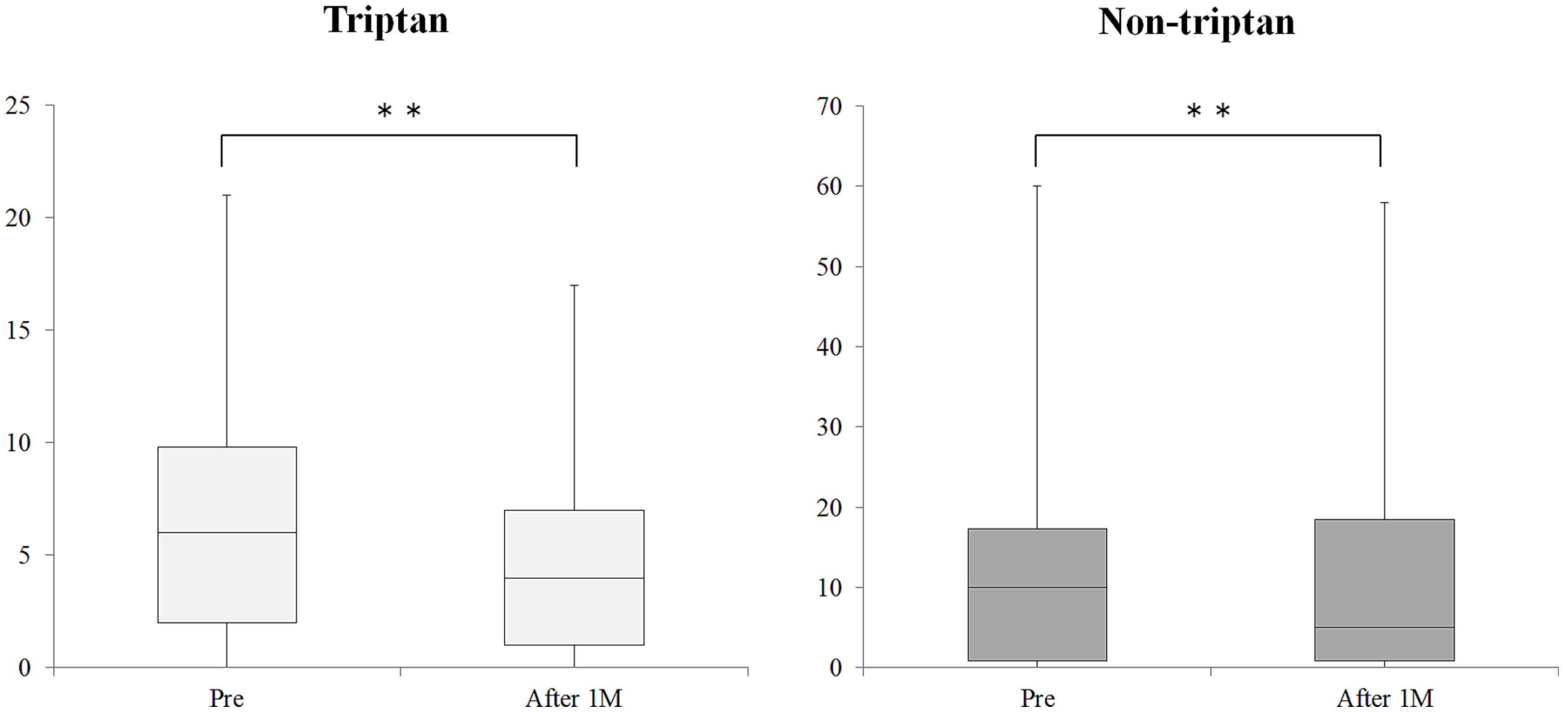
Changes of changes in monthly amounts of triptans and non-triptans before and after Chotosan treatment. The boxes represent the 25 and 75% interquartile ranges, and the line inside the box represents the median. **Significant differences compared with baseline, *p* < 0.01; M, month.

The median MHDs after taking Chotosan, the primary endpoint, was 14.0 [IQR, 7.8–23.8] days, which was significantly decreased from 19.0 [IQR, 14.8–25.8] days at baseline (*p* < 0.001, Figure 2). The absolute change value of MHDs was −4.51 days [95% CI: −5.76, −3.26]. All 27 patients whose MHDs decreased by more than 33% with Chotosan were willing to take and actually continued taking the medication; therefore, the efficacy rate of Chotosan was 52.9% (27/51). Median MMDs also decreased significantly from 3.0 [IQR, 0.0–6.0] days to 1.0 [IQR, 0.0–3.8] days (*p* < 0.05). The absolute change value of MMDs was −1.10 days [95% CI: −2.13, −0.06]. The median amounts of triptan and non-triptan tables decreased significantly from 6.0 [IQR, 2.0–9.8] tablets (*p* < 0.01) to 4.0 [IQR, 1.0–7.0] tablets, and median 10.0 [IQR, 0.8–17.3] tablets to 5.0 [IQR, 0.8–18.5] tablets (*p* < 0.01), respectively (Figure 3). Side effects of Chotosan occurred in 2 patients (malaise 1, itch 1), with an incidence rate of 3.9%.

In a supplementary analysis, when effective was defined as a 50% or greater reduction in MHDs, the efficacy rate of Chotosan was 27.5% (14/51). Three months after starting Chotosan treatment, the median MHDs and MMDs were 11.0 [IQR, 8.0–17.0] days and 0.0 [IQR, 0.0–3.0] days, respectively, and the median amounts of triptan and non-triptan tablets were 4.0 [IQR, 2.0–6.0] tablets and 3.0 [IQR, 0.0–8.0] tablets, respectively. Comparing baseline and 3 months after starting Chotosan treatment, significant reductions were observed in MHDs (*p* < 0.001), MMDs (*p* < 0.01), triptan (*p* < 0.01), and non-triptan (*p* < 0.001). Furthermore, comparing 1 month and 3 months after starting Chotosan treatment, a significant reductions was observed in non-triptan (*p* < 0.01), but no significant differences were observed in MHDs, MMDs, or triptan.

### 3.3 Comparison between effective and ineffective groups

The results of the comparison between the effective and ineffective groups are shown in Table 2. Significant differences were found between the two groups in headache and acute medication, and types of prophylactic medications categories. In the headache and acute medication category, significant differences were found in MHDs (*p* < 0.01) and non-triptan use (*p* < 0.05), respectively. In the types of prophylactic medications category, significant differences were found in anticonvulsants (*p* < 0.001) and antidepressants (*p* = 0.01). There were no significant differences between the two groups in age, sex, onset years, family history of headache, history of psychiatric disorders, migraine with aura, classification of migraine, oral prophylactic medication available, or CGRP-mAb treatment.

**Table 2:** Comparison of the effective and ineffective groups.

	Effective group	Ineffective group	*p*-value
	*n* = 27	*n* = 24	
Age (years), mean ± SD	41.7 ± 10.8	39.3 ± 10.8	0.445
Sex, female; *n* (%)	24 (85.2%)	22 (91.7%)	0.671
Onset years; *n* (%)			0.968
Teens and younger	12 (44.4%)	11 (45.8%)	
20s	10 (37.0%)	8 (33.3%)	
30s	4 (14.8%)	5 (20.8%)	
40s and older	1 (3.7%)	0	
Family history of headaches; *n* (%)	16 (59.3%)	16 (66.7)	0.772
History of psychiatric disorders; *n* (%)	2 (7.4%)	6 (25.0%)	0.127
Migraine with aura; *n* (%)	12 (44.4%)	7 (29.2%)	0.385
Classification of migraine			0.059
EM	10 (37.0%)	3 (12.5%)	
HFEM	10 (37.0%)	6 (25.0)	
CM	2 (7.4%)	4 (16.7)	
Migraine with MOH	5 (18.5%)	11 (45.8%)	
Headache and acute medication			
MHD	15.0 [12.5–20.0]	24.0 [18.0–28.0]	<0.01
MMD	1.0 [0.0–6.0]	3.0 [0.8–5.3]	0.497
Triptan (tablets/month)	6.0 [2.0–11.5]	6.0 [1.8–7.3]	0.327
Non-triptan (tablets/month)	6.0 [0.0–11.5]	15.5 [2.8–21.0]	<0.05
Oral prophylactic available	19 (70.4%)	24 (100.0%)	0.416
Types of prophylactic medications^*^			
Anticonvulsants	15 (55.6%)	23 (95.8%)	0.001
Antidepressants	11 (40.7%)	19 (79.2%)	0.01
Calcium channel blockers	7 (25.9%)	8 (33.3%)	0.759
Beta blockers	0	0	NA
CGRP-mAb treatment	8 (29.6%)	11 (45.8%)	0.261

Data are expressed as median [interquartile ranges] or number (%), CGRP-mAb, anti-calcitonin gene-related peptide monoclonal antibody; CM, chronic migraine; EM, episodic migraine; HFEM, high frequency episodic migraine; MHD, monthly headache days; MMD, monthly migraine days; MOH, medication overuse headache; *n*, number; NA, not available; SD, standard deviation; *Including overlaps.

## 4 Discussion

In the present study, 51 patients with migraine accompanied by TTH were treated with the herbal medicine Chotosan following treatment with Western medications. Chotosan decreased median MHDs significantly from 19.0 [IQR, 14.8–25.8] days to 14.0 [IQR, 7.8–23.8] days, with an efficacy rate of 52.9%. There were also significant reductions in MMDs and monthly amounts of triptan and non-triptan tablet usage. The incidence of side effects from Chotosan was 3.9%. Compared with the ineffective group, the effective group had fewer MHDs before starting Chotosan treatment and had less use of non-triptans, anticonvulsants, and antidepressants.

Herbal medicines are complementary medicines and are less effective than Western medicines. Therefore, to accurately evaluate the effectiveness of herbal medicine “Chotosan,” a unique definition of effectiveness involving both objective and subjective indicators was used. The study analyzed patients with refractory migraine who did not respond to Western medical treatment, and their conditions were similar to those of chronic pain patients. Therefore, as an objective indicator, the cut-off value for MHD reduction was set at 33%, which is used to evaluate improvement in chronic pain (23). As a subjective indicator, the condition that the patients themselves felt the effects of the medication and actually continued taking it was added (16). In a supplementary analysis using the cut-off value for MHD reduction was set at 50%, which is commonly used to evaluate migraine treatments, the efficacy rate of Chotosan dropped to 27.5%. It is important to note that, due to the methodology of this study design, the efficacy rate of Chotosan may be overestimated. Additionally, the results of a supplementary analysis 3 months after starting Chotosan treatment showed significant improvements compared to before treatment. However, these results may include the influence of other newly added medications, and the effectiveness of Chotosan alone may not be properly evaluated.

Of the total 1,030 patients with migraine analyzed in the present study, 896 patients (87%) responded well to treatment with Western medications in line with the Clinical Practice Guideline for Headache (4), demonstrating how effective these treatments are. However, it was found that approximately 10% of patients with migraine did not achieve sufficient therapeutic results with Western medications alone and required the addition of herbal medicines. Of the patients included in the analysis, the proportion of patients with EM was lower than that of all migraine patients (25.5% vs. 59.5%), and the proportions of patients with HFEM (31.4% vs. 19.0%) and migraine with MOH (31.4% vs. 10.2%) were higher. Similarly, the proportion of patients receiving CGRP-mAb treatment was higher in the analyzed patients (37.3% vs. 15.0%). These background findings of analyzed patients were consistent with the treatment policy of prescribing additional herbal medicines to patients who were not obtaining sufficient therapeutic results from Western medications. Patients receiving CGRP-mAb treatment, especially migraine with MOH, have often already tried multiple oral prophylactic medications (9) and, therefore, they have limited treatment options available if frequent headaches recur after CGRP-mAb administration. The herbal medicines, whose mechanisms of action differ from those of Western medications, may be effective treatment options when Western medications do not improve headache symptoms sufficiently.

TTH is the most prevalent form of primary headache in the general population, but paradoxically the least studied headache (24). TTH is characterized by frequency and by a mild to moderate headache that is not associated with the typical debilitating migraine symptoms of nausea, vomiting, photophobia, and phonophobia (21). TTH could often coexist with migraine, but distinguishing between TTH and mild type of migraine without aura can be difficult (24–26). Therefore, the prevalence of coexisting migraine and TTH is difficult to determine and remains unclear. Nonpharmacological treatments are effective for patients with TTH, with physical therapy including postural modification, relaxation, and exercise programs being the most widely used. Non-triptan medications such as acetaminophen and NSAIDs are recommended as acute analgesics for TTH, whereas triptans, muscle relaxants, and opioids are ineffective (24–26). Antidepressants are recommended as pharmacological preventive therapy for TTH, whereas propranolol and valproic acid are ineffective (24–26). Of the patients analyzed in the present study, 58.8% had already been prescribed antidepressants and were taking about 10 tablets of non-triptans per month, indicating that more than half of the patients were already receiving standard Western medications for TTH symptoms. In the present study, patients with migraine accompanied by TTH who were not prescribed antidepressants were unwilling to take antidepressants themselves and preferred herbal medicines instead. For Japanese people, herbal medicines are recognized as having few side effects and being gentle on the body, so there is little psychological resistance to them, and many patients are readily willing to take them in clinical practice.

The present study is the first report showing that Chotosan significantly reduced MHDs, MMDs, and the amount of acute medication usage through statistical analysis of the data collected from patients’ headache diaries. Treatment with Chotosan significantly reduced MHDs, which was likely due to a reduction in TTH symptoms. The reduction in MHDs also resulted in a significant reduction in the monthly amount of non-triptan tablets used for the acute treatment of TTH. Furthermore, it is noteworthy that Chotosan also significantly reduced MMDs, which refers to migraine attacks, thereby reducing the monthly amount of triptan tablets used. It is unclear whether Chotosan has a direct or indirect effect on suppressing migraine attacks. It has been suggested that nitric oxide radicals can be involved in the pathogenesis of chronic headaches (27, 28). Chotosan has the effect of direct nitric oxide radical scavenging activity (29), which may contribute to the reduction of migraine attacks. Chotosan also exerts antidepressant-like effects through changes in the serotonergic and dopaminergic systems, which may contribute to the reduction of TTH symptoms (30). Furthermore, recent animal studies reported the following pharmacological effects of Chotosan. It exerts anxiolytic-like effects in the context of inflammation-induced anxiety, potentially mediated by the inhibition of 5-HT_2A_ receptor hyperfunction (31). It exerts neuroprotection via activating the nuclear factor-E2-related factor 2-mediated antioxidant pathway (32). However, these findings were based on animal studies and not direct human data, so it is unclear how these pharmacological effects actually contributed to the improvement of migraines.

Comparison of the effective and ineffective groups showed significant differences in headache acute medication, and types of prophylactic medications categories. In the headache and acute medication category, MHDs and non-triptan use were significantly higher in the ineffective group. In the types of prophylactic medications category, the ineffective group used more anticonvulsants and antidepressants. These results suggest that Chotosan may be less effective for patients with frequent headaches, who have already tried multiple prophylactic medications, and who regularly take large amounts of non-triptan medications. Although the difference was not significant, the proportion of migraine with MOH was higher in the ineffective group (45.8%) than in the effective group (18.5%), and 45.8% of patients in the ineffective group were already receiving CGRP-mAbs. The gold standard management of MOH consists of abrupt discontinuation of overused medications and initiation of preventive treatment (33–35). Meanwhile, we previously reported that administration of CGRP-mAbs to patients with migraine with MOH reduced the frequency of headache symptoms and acute medication use without abrupt drug discontinuation or hospitalization (9). However, even when CGRP-mAb administration is effective, there are cases in which the number of headache days increases again or TTH persists. In such refractory MOH cases, it is difficult to expect any effect even if herbal medicines are added.

The present study has limitations that should be noted. First, this was a single-center, retrospective, non-randomized, and it did not demonstrate the effectiveness of Chotosan compared with a control group. Second, only 51 of the 1,030 patients were included in the analysis, and because herbal treatments were self-selected by patients, the exclusion process may have introduced bias towards a milder, more compliant subset. Third, the small sample size limited power, generalizability, and absolute effect sizes. Fourth, criteria were not set for the timing of starting Chotosan treatment. Therefore, the efficacy of Chotosan was evaluated in various settings with other oral migraine prophylactic medications or CGRP-mAbs, and these concurrent prophylactic medications may have affected headache frequency. Fifth, non-pharmacological treatments such as physical therapy were not used to treat TTH symptoms. Sixth, the long-term outcomes of Chotosan are still unknown. Seventh, herbal medicines are primarily used in Asia and are only available to a limited extent in countries such as Europe and America. Therefore, the findings of this study are likely to be culture-specific, and their applicability to Western populations is unclear. Finally, because this was a non-randomized drug trial, the results of this study alone are insufficient to prove the usefulness of the herbal medicine.

## 5 Conclusion

Treatment with the herbal medicine Chotosan following treatment with Western medications for migraine accompanied by TTH significantly reduced not only MHDs, but also MMDs, and reduced the monthly amount of triptan and non-triptan tablet usage. The efficacy rate of Chotosan was 52.9%, and the side effect rate was 3.9%. For patients with migraine accompanied by TTH who have not responded adequately to Western medications or who are unwilling to take antidepressants, the herbal medicine Chotosan is a treatment option worth considering.

## Data Availability

The raw data supporting the conclusions of this article will be made available by the authors, without undue reservation.
